# Bevacizumab-Induced Hypertension as a Potential Physiological Clinical Biomarker for Improved Outcomes in Patients With Recurrent Glioblastoma Multiforme: A Systematic Review

**DOI:** 10.7759/cureus.29269

**Published:** 2022-09-17

**Authors:** Kokab Irfan Khan, Prasana Ramesh, Suthasenthuran Kanagalingam, FNU Zargham Ul Haq, Nishok Victory Srinivasan, Aujala Irfan Khan, Ghadi D Mashat, Mohammad Hazique, Safeera Khan

**Affiliations:** 1 Research, California Institute of Behavioral Neurosciences & Psychology, Fairfield, USA; 2 Internal Medicine, California Institute of Behavioral Neurosciences & Psychology, Fairfield, USA; 3 General Surgery, California Institute of Behavioral Neurosciences & Psychology, Fairfield, USA; 4 Pediatrics, California Institute of Behavioral Neurosciences & Psychology, Fairfield, USA

**Keywords:** antiangiogenic agent, vascular endothelial growth factor, recurrent glioblastoma multiforme, hypertension, bevacizumab

## Abstract

With the advancement in medicine leading to the discovery of anti-vascular endothelial growth factor drugs, numerous oncologists are now commonly using antiangiogenic medications to improve outcomes and attain disease control. Thus, the significance of prognostic and predictive indicators in patient selection has become increasingly imperative. These biomarkers have the capacity to be highly effective and can easily be implemented in various diagnostic and therapeutic settings on a large scale. Overall, it has the potential of significantly decreasing mortality in a fatal disease and possibly achieving partial or complete remission. Many clinical trials have shown the efficacy of bevacizumab in treating malignancies. However, there are currently no known predictive or prognostic biomarkers for bevacizumab in glioblastoma multiforme (GBM). Several clinical studies have evaluated bevacizumab-induced hypertension as a potential marker in patients with different malignancies, including recurrent glioblastoma multiforme (rGBM). This systematic review was performed to determine the association of bevacizumab-induced hypertension with outcomes in patients with advanced brain cancer and to assess whether hypertension (HTN) can be used as a prognostic factor. The review was conducted according to Preferred Reporting Items for Systematic Review and Meta-Analysis (PRISMA) guidelines, and the databases were searched from January 2012 to June 2022. This review aimed to evaluate six published studies to investigate the relationship between hypertension and the outcomes of patients with rGBM treated with bevacizumab. Among the included publications, four out of six were retrospective and featured a positive result regarding hypertension being used as an independent predictive factor of survival outcomes in rGBM. However, two studies showed negative results. This can be attributed to the limited subsets of patients and the duration of the studies. In conclusion, bevacizumab-induced hypertension may represent a prognostic factor in patients with rGBM.

## Introduction and background

Glioblastoma multiforme (GBM) is the most common aggressive primary brain cancer, accounting for 54% of all malignant brain tumor cases in adults in the United States alone [1]. It is known as a grade IV diffuse astrocytic and oligodendroglia tumor by the World Health Organization [2]. Tumor progression and recurrence rates remain high in patients with GBM. Despite diagnostic and therapeutic advances, universal fatality is seen in almost all patients. The poor prognosis related to GBM is well documented, with the median survival from the time of diagnosis for most patients being eight months and a five-year survival rate of less than 10% [3], making it emergent to investigate approaches to improve the outcomes of GBM patients.

Antiangiogenic drugs primarily act by inhibiting vascular endothelial growth factors (VEGF), thereby preventing interaction with VEGF receptors on tumor and vascular endothelial cells. These VEGF receptors in GBM are exceedingly expressed, as it is a highly vascular tumor; hence targeting it may potentially reduce tumor vascularization and growth and therefore seems to be a rational therapeutic approach [4,5]. In 2009, Food and Drug Administration (FDA) granted accelerated approval for bevacizumab (Avastin), a humanized anti-VEGF IgG1 antibody, for the treatment of recurrent glioblastoma multiforme (rGBM) [6].

Bevacizumab can effectively increase the objective response rate and median progression-free survival in patients with rGBM [7]. However, hypertension (HTN) is a common adverse event of bevacizumab treatment [7]. To our knowledge, the underpinning physiology for this side effect is not fully understood. It is hypothecated to be secondary to inhibition of the VEGF signaling pathway, which triggers an increase in systemic vascular resistance due to the decreased production of nitric oxide in endothelial cells [8,9]. HTN is usually treated by standard anti-hypertensive therapy. Interestingly, many clinical studies have found improved clinical response and survival outcomes for those patients who develop HTN secondary to bevacizumab treatment in metastatic breast, non-small cell lung, colorectal, renal, and ovarian cancer [5,6,9-14].

Throughout treatment for GBM, hypertension severity can be assessed objectively and thus may be beneficial when making an early decision on whether to alter the course of disease treatment. The potential advantages of such a predictor include estimating the efficacy and activity of anti-VEGF agents in patients with rGBM. However, although many potential prognostic markers of bevacizumab have been reported, to the best of our knowledge, there is no definite predictor of improved outcomes in rGBM patients. Thus, the purpose of this study was to perform a systematic review to determine if the occurrence of bevacizumab-induced hypertension is a prognostic factor of response and survival in patients with rGBM.

## Review

Methods

The Preferred Reporting Items for Systematic Review and Meta-Analysis (PRISMA) 2020 guidelines were used for conducting this systematic review [15].

Search Sources and Strategy

An extensive search of PubMed, PubMed Central (PMC), ScienceDirect, and Google Scholar was performed for literature published between 2012-2022 to retrieve relevant literature that reported hypertension progression or response and survival in recurrent glioblastoma multiforme patients treated with bevacizumab. The study was executed using a pre-specified search strategy with strict eligibility criteria. The combination of search terms used was "Avastin", "bevacizumab", "hypertension", and "glioblastoma multiforme" in all fields. Reference lists from the articles were also examined for additional suitable studies.

Eligibility Criteria

Firstly, duplicates were screened and removed using EndNote (Clarivate, Philadelphia, Pennsylvania). Then, screening of the remaining papers was done based on title and abstracts. The full text of the results was then evaluated based on the quality of the article. Only related articles that fulfilled >60% of the assessment criteria in the quality appraisal were included. Of the selected articles, only those published in English from 2012-2022 were included. Also, the focus was on the human adult population (>18 years). The papers excluded from this review involved the pediatric population, gray literature, and animal studies.

Risk Bias Assessment

Newcastle-Ottawa Scale was used to assess the quality of studies for risk of bias, and only articles that met >60% of the evaluation parameters were included. The following three factors were measured to score the quality of included studies: (1) selection, (2) comparability, and (3) outcome.

Results

**Figure 1:**
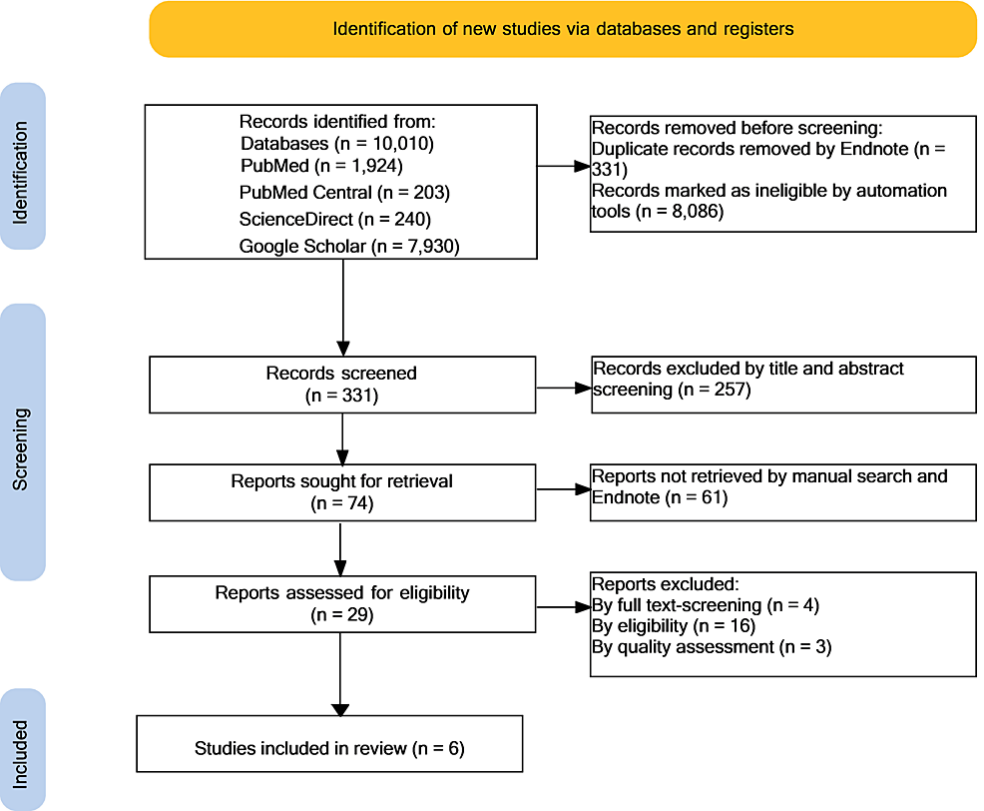
Comprehensive Preferred Reporting Items for Systematic Review and Meta-Analysis (PRISMA) flowchart n: number of records

Ten thousand ten results were identified from the databases described above. Eight thousand four hundred seventeen results were removed because of duplicates (331 results) and automation tools (8,086 results). A further 257 were excluded by screening the title and abstract. Seventy-four remaining were sought for retrieval, but 61 could not be retrieved. Twenty-nine reports were evaluated for eligibility, and 23 were removed after screening for full text, English language only papers, and quality assessment. The PRISMA flowchart (Figure 1) demonstrates the filtering process: the final criteria were met by six articles. The individual characteristics of the studies and outcomes included in this systematic review are presented in Table 1.

**Table 1:** Summary and characteristics of the studies included in this review HTN: hypertension; rGBM: recurrent glioblastoma multiforme; CTCAE: Common Terminology Criteria for Adverse Events; vs.: versus

Author and year of publication	Purpose of study	Type of study	Location	Number of patients	Conclusion
Carvalho et al., 2020 [16]	Assessment of whether bevacizumab-induced hypertension and proteinuria result in higher survival as compared to patients that do not have them.	Retrospective study	Portugal	140	Bevacizumab therapy resulting in HTN and proteinuria is correlated to increased survival outcomes and is a possible indicator of active anti-tumor effect.
Liau et al., 2018 [17]	Assessment of the efficacy and safety profile of bevacizumab in patients with rGBM to determine whether their response differed from that reported in other clinical trials, and to examine potential prognostic factors for survival.	Retrospective study	Taiwan	76	Provides evidence that gender, performance status, and bevacizumab-induced hypertension may be predictive clinical markers for survival outcomes in rGBM patients.
Bumes et al., 2016 [18]	Comparison of arterial HTN measurement using CTCAE version 3.0 vs. 4.0 in the bevacizumab trial.	Retrospective study	Switzerland	44	Demonstrated that arterial HTN has been previously underestimated in clinical trials in glioma patients. CTCAE version 3.0 underreported the incidence and grade of bevacizumab-induced hypertension within clinical trials.
Zhong et al., 2015 [19]	Determining differential response to bevacizumab therapy inducing hypertension in patients with rGBM.	Retrospective study	United States of America	82	Compared to normotensive patients, there is a significant improvement in survival outcomes in patients who develop bevacizumab-induced hypertension.
Wagner et al., 2014 [20]	Assessment of the role of HTN as a potential predictive marker for bevacizumab efficacy in rGBM.	Prospective study	Switzerland	40	Does not support the notion of bevacizumab-induced hypertension as a predictor of clinical benefit in high-grade gliomas.
Lombardi et al., 2013 [21]	Evaluation of a possible association between outcome and onset of HTN in patients with rGBM treated with angiogenesis inhibitors.	Retrospective study	Italy	53	Association between HTN onset and improved clinical outcome in rGBM patients receiving antiangiogenic drugs is possible.

Discussion

Timing of Onset of Hypertension After Treatment Initiation

An objective analysis of five of the six studies was done to find the time of onset of hypertension after bevacizumab therapy was initiated. Carvalho et al. reported the longest median time of 15 weeks [16]. Similarly, the study by Wagner and colleagues suggested 13 weeks, whereas Zhong et al. stated three weeks as the shortest median time of onset [19,20]. Lombardi et al. and Liau et al. reported 7.8 weeks and 9.6 weeks, respectively [17,21].

Grading of Hypertension

We analyzed a total number of 435 patients, of which 195 (44.8%) developed treatment-related hypertension; the majority fell in grade 2 and grade 3 criteria enlisted by Common Terminology Criteria for Adverse Events (CTCAE). The studies included in this review used National Cancer Institute - Common Toxicity Criteria scale version 3.0, 4.0, and 5.0. On the breakdown of the studies, four used version 4.0, one used version 5.0, and one used versions 3.0 and 4.0 as they compared the efficacy of each type. Below, Table 2 summarizes and compares the three versions of CTCAE for hypertension in adults [22].

**Table 2:** Overview of CTCAE versions 3.0, 4.0, and 5.0 CTCAE: Common Terminology Criteria for Adverse Events; hrs: hours; BP: blood pressure; HTN: hypertension

CTCAE adult version	Grade 1	Grade 2	Grade 3	Grade 4	Grade 5
3.0 [22]	Asymptomatic, transient (<24 hrs) diastolic increase by >20 mmHg or >150/100 mmHg	Recurrent or persistent (≥24 hrs) or symptomatic increase by >20 mmHg (diastolic) or >150/100	Requiring more than one drug or more intensive therapy than previously needed	Life-threatening consequences: hypertensive crisis	Death
4.0 [22]	Prehypertension: systolic BP is 120-139 mmHg or diastolic BP is 80-89 mmHg	Stage 1 HTN: (a) systolic BP is 140-159 mmHG or diastolic BP is 90-99 mmHg; (b) medical intervention indicated; (c) recurrent or persistent (≥24 hrs), symptomatic diastolic BP increase by >20 mmHg or >140/90 mmHg	Stage 2 HTN: (a) systolic BP ≥160 mmHg or diastolic BP is ≥100 mmHg); (b) medical intervention is indicated; (c) more than one drug or more intensive therapy than previously used indicated	Life-threatening consequences: (a) malignant hypertension, transient or permanent neurologic deficit, hypertensive crisis; (b) urgent intervention indicated	Death
5.0 [22]	Systolic BP is 120-139 mmHg or diastolic BP is 80-89 mmHg	(a) Systolic BP is 140-159 mmHg or diastolic BP is 90-99 mmHg if previously in normal limits; (b) change in baseline medical intervention indicated; (c) recurrent or persistent (≥24 hrs); (d) symptomatic diastolic increase by 20 mmHg or >140/90 mmHg; (e) monotherapy indicated or initiated	(a) systolic BP ≥160 mmHg or diastolic BP is ≥100 mmHg; (b) medical intervention indicated; (c) more than one drug or more intensive therapy than previously used indicated	Life-threatening consequences: (a) malignant hypertension, transient or permanent neurologic deficit, hypertensive crisis; (b) urgent intervention indicated	Death

Based on the data available, outcomes and responses were assessed by either a comparison between normotensive (grade 0) and all grades of hypertension (1-4), or a comparison between low-grade hypertension (0-1) and high-grade hypertension (2-4). Below, Table 3 summarizes the analysis of the grading of hypertension found using CTCAE versions, time of onset of HTN after treatment initiation, bevacizumab regimen used, and the number of patients identified with treatment-related HTN.

**Table 3:** Summarizes treatment-related hypertension characteristics and bevacizumab regimen used HTN: hypertension; CTCAE: Common Terminology Criteria for Adverse Events; TMZ: temozolomide; NA: not assessed; x: data not given; vs.: versus; +: and; ±: and/or

Author name	Treatment agent	Total number of patients	Time of onset of HTN	Number of patients with treatment-related HTN	CTCAE version	Grade 1	Grade 2	Grade 3	Grade 4	Grade ≥ 3
Carvalho et al. [16]	All patients were treated with second-line bevacizumab-based therapy: (a) bevacizumab (10 mg/kg); (b) bevacizumab (10 mg/kg) + irinotecan (340 or 125 mg/m^2^) every two weeks; or (c) bevacizumab (10 mg/kg) every two weeks + lomustine (90 mg/m^2^) every six weeks	140	15 weeks	23	5.0	1	8	12	1	x
Liau et al. [17]	Bevacizumab (10 mg/kg) every 14 days	76	68 days	70	4.0	x	x	x	x	37 grade >3
Bumes et al. [18]	Bevacizumab (10 mg/kg) on days 1 and 15 + TMZ (40 mg) daily in 28-day cycles	44	NA	11 (with CTCAE 3.0) vs. 35 (with CTCAE 4.0)	3.0 and 4.0	0 vs. 5	7 vs. 11	4 vs. 19	x	4 vs. 19
Zhong et al. [19]	(a) Bevacizumab (5 mg/kg or 10 mg/kg) every 14 days, (b) ± irinotecan (125 mg/m^2^) every two weeks; (c) combined bevacizumab and TMZ (150 mg/m^2^ to 200 mg/m^2^ on days 1 to 5 of a 28-day cycle) or daily metronomic administrations of 50 mg/m^2^	82	21 days	30	4.0	x	17	13	x	x
Wagner et al. [20]	Bevacizumab (10 mg/kg) every 21 days	40	3 months	17	4.0	x	10	7	x	x
Lombardi et al. [21]	Bevacizumab (10 mg/kg) ± irinotecan every two weeks; oral sorafenib (400 mg) twice a day + TMZ every day	53	1.8 months	20	4.0	x	12	8	x	x

In 2020, Carvalho et al. studied 140 patients, of which 23 developed bevacizumab-induced hypertension, resulting in the following grades: grade 1 - one; grade 2 - eight; grade 3 - 12; grade 4 - one [16]. Similarly, in 2018, Liau et al. assessed 76 patients, of which 70 developed hypertension, out of which 37 patients developed grade 3 HTN [17]. Moreover, in 2015, Zhong and colleagues found that out of 82 patients, 30 got hypertension, with the majority reported in grades 2 and 3, containing 17 and 13 patients, respectively [19]. Also, in 2014, Wagner et al. studied 40 patients, of which 17 resulted in hypertension, with the majority falling in grade 2 - 10 patients, followed by seven patients in grade 3 [20]. In 2013, Lombardi and colleagues assessed 53 patients, of which 20 developed HTN, with the following grades: grade 2 - 12; and grade 3 - eight. Additionally, 16 patients had systolic-defined and diastolic-defined HTN, and four had only diastolic-defined HTN [21]. Bumes et al., in 2016, studied 44 patients. When using version 3.0, they categorized the patients into the following grades: grade 2 - seven; grade 3 - four, grade ≥3 - four, whereas, when using version 4.0, they categorized the patients into the following grades: grade 1 - five, grade 2 - 11, grade 3 - 19, grade ≥3 - 19. Hence, proving that CTCAE version 3.0 may underreport the actual incidence of bevacizumab-induced hypertension within clinical trials [18]. 

Additionally, the study by Bumes and colleagues showed that version 4.0 detected HTN and its events with a significantly higher sensitivity than CTCAE version 3.0 in glioma patients treated with bevacizumab [18]. But to our knowledge, no study compares the efficacy of CTCAE version 5.0 and version 4.0. Hence, further studies would be needed to prove the sensitivity of the latter versions in reporting the incidence and grade of bevacizumab-induced hypertension within clinical trials.

Median Progression-Free Survival in Hypertensive Versus Non-Hypertensive Patients

In four of the total studies assessed, the primary outcome was progression-free survival (PFS), defined as the time between arbitrary assignment and any progression or death from any cause concerning the severity of hypertension in patients given bevacizumab. The median PFS was analyzed between normotensive and hypertensive patients. Carvalho et al. reported a significant difference between the normotensive and hypertensive groups (p=0.005) [16]. The median PFS is four and 12 months, respectively [16]. Zhong et al. showed similar significant results with a median PFS of 2.5 months in normotensive and 6.7 months in hypertensive cases (p<0.001) [19]. Lombardi et al. also reported a minor insignificant variance in median PFS in both normotensive and hypertensive groups, which was 2.1 and 4.1 months, respectively [21]. However, Lombardi and colleagues reported no significant association between hypertension and median PFS compared to the other two studies [21]. Yet, they did report a significant association between hypertension and six-month PFS, which was 32% (p=0.03) [21]. Hence, bevacizumab-induced hypertensive patients showed a substantial difference in PFS compared to the normotensive patients on bevacizumab for rGBM. Furthermore, Liau et al.'s study found that the patients who developed grade 3 hypertension had better PFS than those that did not [17]. The median PFS for grades 0-2 was found to be 3.4 months; for patients with grade 3 HTN, it was 7.8 months (p<0.001) [17].

Median Overall Survival in Hypertensive Versus Non-Hypertensive Patients

In four of the five studies evaluated for this topic, the secondary outcome was overall survival (OS), defined as the time between initiation of bevacizumab and death due to any cause, with hypertension as a predictor. The median OS was analyzed between normotensive and hypertensive patients. Carvalho et al. reported a significant difference between the normotensive and hypertensive groups (p=0.035) [16]. The median OS was 18.0 months and 27.0 months, respectively [16]. Zhong et al. presented similar statistically significant results initially, with a median OS of 4.9 months in normotensive and 11.7 months in hypertensive cases (p<0.001) [19]. However, after adjustment of guarantee-time bias, with a landmark time chosen as 2.5 months, the median OS reported was 3.6 months for normotensive and 9.2 months for hypertensive patients [19]. Lombardi et al. likewise reported a marked difference in median OS in both normotensive and hypertensive groups, which was 4.8 and 9.8 months, respectively (p=0.001). The study reported a significant association between hypertension and disease control rate (p=0.04) [21]. However, in one of the studies where the primary outcome was OS, Wagner et al. reported in their prospective analysis that normotensive patients had a better median OS of 9.0 months compared to 5.8 months for hypertensive patients [20]. Overall, majority of the studies demonstrated a substantial increase in median OS as compared to the normotensive patients on bevacizumab for rGBM.

Liau and colleagues found that the patients who developed grade 3 hypertension had better OS than those that didn't develop grade 3 hypertension. The median OS for grades 0-2 was found to be 3.6 months; for patients with grade 3 hypertension, it was 9.7 months. This study also reported an overall response rate of 59.2%, which included 19 patients with a complete response and 26 with a partial response [17].

Furthermore, Bumes et al. showed no significant difference in PFS and OS in patients with hypertensive events compared to patients without hypertensive events in both classifications (CTCAE version 3.0 and version 4.0) [18].

Bevacizumab Monotherapy Versus Combination Treatment-Related Hypertension and Outcomes

On further analysis of single-agent bevacizumab and multi-drug regimen of bevacizumab-induced hypertension, we found that Carvalho and colleagues reported no statistical difference in survival data between different bevacizumab-based regimens [16]. Of the 23 patients who developed treatment-related hypertension, two were on bevacizumab monotherapy, 10 were on bevacizumab and irinotecan therapy, and 11 were on bevacizumab and lomustine [16]. Similarly, Lombardi et al. reported no significant difference in disease control, PFS, OS, and hypertension onset in terms of different antiangiogenic treatments [21]. In contrast, the median OS and PFS for patients treated with sorafenib were 7.4 and 3.3 months, versus bevacizumab's 6.3 and 2.4 months [21]. However, studies with a large sample size are needed to more effectively compare sorafenib-induced HTN with bevacizumab-induced HTN in rGBM. Moreover, Zhong and colleagues found no significant difference in clinical outcomes regarding the type of systemic therapy regimen received (p=0.31) between normotensive and hypertensive patients [19]. Yet, the hypertensive group did receive significantly more doses of bevacizumab compared with the normotensive group (median of 15 doses versus six doses respectively, p<0.01), most likely due to better responses to therapy among the hypertensive patients [19].

Other Prognostic Factors in Assessing Bevacizumab Efficacy in rGBM

We further analyzed additional factors which can gauge the response of bevacizumab in rGBM. Liau et al. found that the female gender was linked to a statistically significant improvement (p<0.001) in median PFS and OS at six and 13.2 months, respectively [17]. However, in the study by Carvalho et al., gender had no statistical impact on OS [16].

Moreover, Liau et al. noted that patients with Eastern Cooperative Oncology Group (ECOG) performance status (PS) ≤1 had higher median PFS and OS than those with ECOG PS >1 [17]. Similarly, Lombardi and colleagues found that ECOG PS was independently associated with prolonged survival (p=0.001, HR=8.9) [21]. However, Carvalho et al. found that ECOG PS had no statistical impact on OS [16].

In the study by Carvalho and colleagues, proteinuria was found to be an independent prognostic factor of PFS and OS and associated with prolonged disease control [16]. The median timing of onset of proteinuria was 18 weeks after initiation of antiangiogenic treatment [16]. Patients with proteinuria had a PFS of 10 months versus four months in patients without proteinuria (p=0.002) [16]. Moreover, treatment-related proteinuria (p=0.018, HR=0.483) was identified as an independent prognostic factor significantly associated with increased OS [16]. However, Liau et al. found no correlation between improved survival and proteinuria [17].

Additionally, several temozolomide (TMZ) cycles >6 (p<0.001, hazard ratio (HR)=0.48) and the beginning of third-line chemotherapy (p=0.016, HR=0.64) as independent prognostic factors were associated with significantly higher OS [16]. Also, isocitrate dehydrogenase (IDH) status was associated with longer survival (p=0.01, HR=6.25) while methylguanine methyltransferase (MGMT) status was not correlated with a longer PFS and OS [21]. However, in another study, MGMT promoter methylation and IDH1 mutation status did not significantly influence PFS and OS [18]. In contrast, Zhong and colleagues found that MGMT status was independently predictive of improved OS [19].

Limitations

Our study contained five retrospective analyses and one prospective analysis. Large-scale studies are needed to prove bevacizumab-induced hypertension as a definite predictive factor in rGBM. Two studies had a good subset of patients but were only abstracts and therefore excluded from this study. We were also limited to our selection criteria, which specified a timeline of ten years and exclusive English literature only. Also, since the fatality rate of GBM is so high, most of these patients may have shown limited outcomes before succumbing to the disease. Hypertension is a common clinical presentation. While the aim was to focus on bevacizumab-induced hypertension publications (reflected in the keyword strategy), literature may exist that investigates this patient profile without explicitly referencing it. Simply put, this review has high specificity but possibly low sensitivity. 

## Conclusions

We noticed a variance in results. Hence, caution is undoubtedly needed before we conclude that bevacizumab-induced hypertension is a reliable clinical marker for the early screening and diagnosis of patients with rGBM. This is due to the limited available data and our study's relatively small sample size. However, our results are a foundation for studies with larger sample sizes and multiple centers, which can further elucidate the correlation between HTN and rGBM in patients treated with bevacizumab. We also suggest more studies to investigate the relationships of other potential prognostic factors (female gender, ECOG PS, proteinuria, IDH1, MGMT status, number of TMZ cycles, and bevacizumab combination therapy) to better gauge the maximum effect of bevacizumab on malignancies. From prior literature, we know that antiangiogenic drugs commonly cause hypertension. Hence, we advise future studies to focus on creating an updated universal guideline for grading, diagnosis, and management of bevacizumab-related hypertension. Also, current studies on potential predictive biomarkers of bevacizumab are either retrospective or adjunct to efficacy and safety trials, and only a few prospective trials have focused on predictive biomarkers. These have the potential to be highly effective and can easily be implemented in various diagnostic and therapeutic settings on a large scale.
